# Trends and Disparities in Ventricular Tachycardia‐Related Mortality According to Cardiomyopathy Type in the United States

**DOI:** 10.1111/jce.70116

**Published:** 2025-09-29

**Authors:** Mohammad Ali Sheffeh, Jeanne du Fay de Lavallaz, Andres Estrada Magana, Konstantinos C. Siontis, Jackson J. Liang

**Affiliations:** ^1^ Department of Internal Medicine Henry Ford Warren Hospital Warren Michigan USA; ^2^ Department of Cardiovascular Medicine Mayo Clinic Rochester Rochester Minnesota USA; ^3^ Department of Clinical Electrophysiology, Frankel Cardiovascular Center University of Michigan Ann Arbor Michigan USA

**Keywords:** cardiomyopathy, disparities, ventricular tachycardia

## Abstract

**Background:**

Recent data show increased ventricular tachycardia (VT) related mortality. We aimed to investigate the trends and disparities of VT‐related mortality according to cardiomyopathy subtypes.

**Methods:**

Mortality and demographic data were obtained from the CDC Wide‐ranging Online Data for Epidemiologic Research database between 1999 and 2020. VT‐related mortality was defined as the underlying cause of death and ischemic cardiomyopathy (ICM) or nonischemic cardiomyopathy (NICM) as the contributing cause of death. The direct method of standardization was utilized to estimate age‐adjusted mortality rates (AAMRs). Temporal trends were evaluated using log‐linear regression models.

**Results:**

A total of 15 888 deaths were related to both VT and ICM, and 16 777 were due to both VT and NICM. There was a significant increase in VT and ICM‐related mortality between 2006 and 2020 with an APC of +1.38% (*p* < 0.05). Similarly, VT and NICM‐related mortality increased between 2008 and 2020 with an APC of +0.60% (*p* < 0.05). ICM had a higher AAMR in males [6.23 (6.12–6.34)], Whites [3.49 (3.43–3.54)], Hispanics [2.11 (1.95–2.26)], and the Midwest region [3.73 (3.61–3.85)] compared to NICM. In contrast, NICM had a higher AAMR in females [1.57 (1.52–1.61)], Black or African Americans [5.02 (4.84–5.20)], and the South region [3.10 (3.03–3.18)]. *p* for all trend < 0.05.

**Conclusions:**

Real‐world data show significant differences in VT‐related mortality according to cardiomyopathy subtypes with prominent sex, race, and regional disparities. Clinical and public health strategies are needed to address inequities and improve outcomes.

## 1 Introduction

Ventricular tachycardia (VT) carries a high risk of morbidity and mortality and can occur in the setting of both ischemic (ICM) and nonischemic cardiomyopathy (NICM). These subtypes differ fundamentally in their underlying arrhythmogenic substrates. ICM is characterized by infarct‐related scar‐based reentry that is often subendocardial, whereas NICM often involves midmyocardial and/or epicardial substrate. A pooled analysis of primary‐prevention ICD trials showed that patients with ICM had a greater risk of all‐cause mortality compared with NICM [1]. A contemporary population‐level analysis showed increased VT‐related mortality since 2007 [2]. Comparative population‐level analyses investigating mortality trends in VT patients with ICM versus NICM are limited. In this study, we analyzed national trends in VT‐related mortality stratified by cardiomyopathy subtype.

## 2 Methods

We queried the CDC Wide‐ranging Online Data for Epidemiologic Research database from 1999 to 2020 to obtain mortality and demographic data [3]. Multiple cause‐of‐death was utilized to identify VT as the underlying cause of death using the International Classification of Diseases‐10th Revision (ICD‐10) codes (I47.2) as previously published [2]. We identified ICM and NICM as the contributing cause of death using ICD‐10 codes (ICM I25.2, I25.5, and NICM I42.0, I42.1, I42.2, I42.3, I42.5, I42.6, I42.7, I42.8, I42.9). We queried each category (ICM and NICM) separately and treated them as distinct groups. We estimated age‐adjusted mortality rates (AAMRs) for a population of 1 000 000 (standardized to the year 2000 population) using the direct method of standardization. Log‐linear regression models were used to analyze temporal trends using the Joinpoint Regression Program of the National Cancer Institute [4]. The Institutional Review Board was exempted due to the deidentified and public nature of the data.

## 3 Results

**Figure 1:**
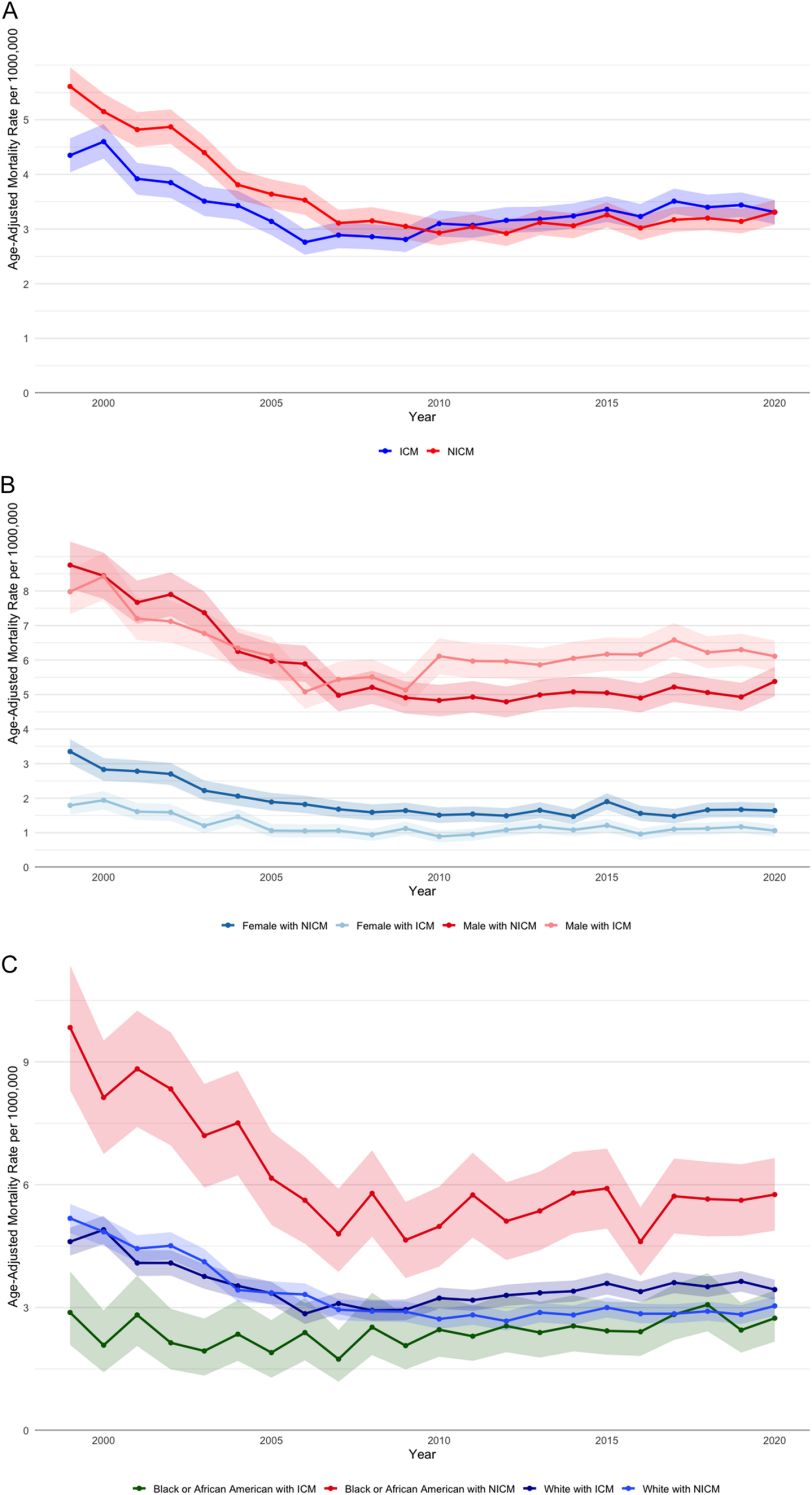
(A) Trends in age‐adjusted mortality rates from ventricular tachycardia between 1999 and 2020 stratified by cardiomyopathy subtypes. (B) Trends in age‐adjusted mortality rates from ventricular tachycardia between 1999 and 2020 stratified by cardiomyopathy subtypes and sex. (C) Trends in age‐adjusted mortality rates from ventricular tachycardia between 1999 and 2020 stratified by cardiomyopathy subtypes and race.

Between 1999 and 2020, a total of 55 317 623 deaths occurred. Of these deaths, 123 945 were solely related to VT, 1 047 008 were related to NICM, and 611 149 were related only to ICM. Additionally, 16 777 deaths were linked to both VT and NICM, while 15 888 deaths involved both VT and ICM. For VT and ICM, there was a significant decline in mortality from 1999 to 2006 with an annual percent change (APC) of −6.58% (*p* < 0.05), followed by a significant increase from 2006 to 2020 with an APC of +1.38% (*p* < 0.05). For VT and NICM, mortality declined from 1999 to 2008 with an APC of −6.81% (*p* < 0.05), followed by a modest but significant increase from 2008 to 2020 with an APC of +0.60% (*p* < 0.05) (Figure 1A).

Males with ICM had a higher AAMR compared to males with NICM [6.23 (6.12–6.34) vs. 4.71 (4.63–4.80)]. In contrast, females with NICM had a higher AAMR compared to females with ICM [1.57 (1.52–1.61) vs. 1.19 (1.15–1.24)] (Figure 1B). Whites with ICM exhibited a higher AAMR compared to NICM [3.49 (3.43–3.54) vs. 2.72 (2.67–2.76)]. Similarly, Hispanics with ICM had a higher AAMR compared to NICM [2.11 (1.95–2.26) vs. 1.88 (1.75–2.01)]. Conversely, Black or African Americans with NICM had a higher AAMR compared to ICM [5.02 (4.84–5.20) vs. 2.41 (2.26–2.55)] (Figure 1C). ICM showed the highest AAMR in the Midwest region [3.73 (3.61–3.85)] compared to other regions, with NICM having the highest AAMR in the South region [3.10 (3.03–3.18)] compared to other areas. In metropolitan regions, NICM had a higher AAMR compared to ICM [3.55 (3.49–3.60) vs. 3.29 (3.24–3.35)]. *p* for all trend < 0.05. In contrast, ICM in nonmetropolitan regions had similar AAMR compared to NICM [3.58 (3.45–3.71) vs. 3.46 (3.33–3.59)], *p* = 0.2.

## 4 Discussion

Nationwide data from 1999 to 2020 demonstrate that VT mortality is significantly influenced by cardiomyopathy subtype and demographic factors. ICM‐related deaths predominate among men, Whites, and Hispanics, whereas NICM‐related deaths are more prevalent among women and Black Americans. These subtype‐specific disparities persist even after age adjustment, underscoring distinct biological and socioeconomic contexts associated with VT mortality.

These findings expand on previous research. Ibrahim and colleagues showed an overall rise in the VT‐related AAMR in patients with underlying cardiovascular disease, with higher mortality rates in males and black patients [2]. Narins and colleagues demonstrated that among ICD recipients, ICM is associated with higher arrhythmic and total mortality compared to NICM. However, our population‐level analysis indicates that NICM mortality remains relatively more prevalent in women and Black individuals [1]. When examining racial health disparities, Black Americans are affected by multiple biological and nonbiological factors, including a higher prevalence of hypertensive heart disease, chronic kidney disease, obesity, and genetic variants associated with arrhythmia risk, and systemic inflammation [5]. Beyond disease phenotype, multiple care‐pathway gaps contribute to these disparities. Black patients remain significantly less likely to undergo ICD implantation, are often referred later for advanced arrhythmia care, and have lower utilization of therapies such as VT ablation. These patterns are well‐documented and reflect a mixture of patient, provider, and health system barriers [6, 7]. To address these gaps, proposed strategies include structured patient navigation programs to improve linkage to subspecialty care, community outreach and education to increase awareness of device‐based prevention and expanding access to device clinics and electrophysiology services in underserved communities.

The observed temporal variations in VT‐related mortality may, in part, be explained by advancements in VT detection over the years. Increased utilization of cardiac implantable electronic devices, the marked rise in implantable loop recorder placement, and improved in‐hospital monitoring likely enhanced the identification of VT events. These factors could account for the recent rise in VT‐related deaths following an initial decline, as improved diagnostic capabilities may have uncovered cases that previously went undetected. This should, however, not impact the sex or race comparison each year, as the same diagnostic criteria were available each year for all subgroups.

While our comprehensive, nationwide analysis provides substantial evidence, several limitations of our study must be acknowledged. Death certificate data may misclassify VT and cardiomyopathy subtypes. Furthermore, the absence of detailed data on clinical metrics such as ejection fraction, device therapy, and socioeconomic status limits our assessment. Moreover, CDC WONDER does not allow granular identification of death certificates that include both ICM and NICM as a contributing cause of death. Although this may slightly influence absolute counts, it is unlikely to affect the overall trends observed. Finally, despite known differences in arrhythmic profile between hypertrophic cardiomyopathy (HCM) and dilated or inflammatory cardiomyopathy, the small proportion of patients who were included due to a diagnosis of HCM (322 patients) did not allow for a meaningful separate subgroup analysis. Prospective studies incorporating these detailed clinical and social metrics are necessary. Initiatives aimed at tracking disparity metrics, expanding electrophysiology services, and engaging affected communities can help bridge existing gaps. Ultimately, developing risk models that integrate sex and ethnicity, alongside ensuring equitable access to life‐saving treatments, is crucial to addressing the unequal burden of VT mortality highlighted by this study.

This study demonstrates significant differences in VT‐related mortality based on cardiomyopathy subtype, underscoring the necessity of considering ischemic and nonischemic etiologies separately. Additionally, prominent sex‐ and race‐based disparities highlight the importance of tailored clinical and public health strategies to address inequities and improve outcomes across diverse patient populations.

## Disclosure

The authors have nothing to report.

## Data Availability

The data that support the findings of this study are available from the corresponding author upon reasonable request.
